# Reply to Oren et al., “New Phylum Names Harmonize Prokaryotic Nomenclature”

**DOI:** 10.1128/mbio.02323-22

**Published:** 2022-09-15

**Authors:** Gaurav Sharma, Salim T. Islam, Praveen Rahi, Mark W. Silby, Donato Giovannelli, Paula V. Welander, Monika Ehling-Schulz, Vasvi Chaudhry, Evelyn Molloy, Christian Hertweck, Sunil Mundra, Vipin Chandra Kalia, Rup Lal, Yogendra Singh, Edward Ruby, Christoph Weigel, Roberto Kolter

**Affiliations:** a Institute of Bioinformatics and Applied Biotechnology (IBAB), Bengaluru, Karnataka, India; b Institut National de la Recherche Scientifique (INRS), Centre Armand-Frappier Santé Biotechnologie, Université du Québec, Institut Pasteur International Network, Laval, Quebec, Canada; c Institut Pasteur, Université Paris Cité, Biological Resource Center of Institut Pasteur (CRBIP), Paris, France; d Department of Biology, University of Massachusetts Dartmouth, Dartmouth, Massachusetts, USA; e Department of Biology, University of Naples “Federico II”, Naples, Italy; f Department of Earth System Science, Department of Biology, Stanford University, Stanford, California, USA; g Institute of Microbiology, Department of Pathobiology, University of Veterinary Medicine, Vienna, Austria; h The Center for Plant Molecular Biology, University of Tübingen, Tübingen, Germany; i Department of Biomolecular Chemistry, Leibniz Institute for Natural Product Research and Infection Biology, Hans Knöll Institute, Jena, Germany; j Department of Biology, College of Science, United Arab Emirates University (UAEU), Al Ain, Abu Dhabi, United Arab Emirates; k Khalifa Center for Genetic Engineering and Biotechnology, United Arab Emirates University, Al Ain, United Arab Emirates; l Department of Chemical Engineering, Konkuk University, Seoul, South Korea; m The Energy and Resources Institute, New Delhi, India; n Delhi School of Public Health, Institution of Eminence, University of Delhi, Delhi, India; o Division of Biology and Biological Engineering, California Institute of Technology, Pasadena, California, USA; p Institute of Biotechnology, Technische Universität Berlin, Berlin, Germany; q Department of Microbiology, Harvard Medical School, Boston, Massachusetts, USA; The Ohio State University

**Keywords:** taxonomy, nomenclature, systematics, classification, International Code of Nomenclature of Prokaryotes (ICNP), polyphasic taxonomy, archaea, bacteria, ICSP, phylum name

## REPLY

We thank Oren et al., 2022 (1) for providing the International Committee on Systematics of Prokaryotes (ICSP) Executive Board's response to the recent opinion piece “Harmonizing Prokaryotic Nomenclature: Fixing the Fuss over Phylum Name Flipping” (2). Herein, we (a group of concerned microbiologists) offer several additional comments on the naming of prokaryotic phyla. Our concerns arise from the perception that the ICSP Executive Board is not directly addressing the plight of microbiologists around the world concerning the long-lasting confusion that the proposed phylum name changes will create throughout the scientific literature. Instead of selectively pointing out certain ICSP rules, we would have appreciated opening a door for a more inclusive discussion about the process of naming phyla. As previously stated (2), we are in favor of the International Code of Nomenclature of Prokaryotes (ICNP) rules for the recognition of the rank of “phylum” and the usage of “-ota” as the suffix for naming phyla. However, we feel that the drastic name changes of six phyla at the prefix level will create significant dissonance and confusion between the old and new names when considering past and future publications.

Oren et al. mentioned (1, 3) that “The proposal of these names (4) was not an action of the ICSP, but the relevant rules of the ICNP were followed correctly.” However, the question is why these names were not proposed directly by the ICSP or under the umbrella of the ICSP itself. How can the same lead author ensure independence between such obvious conflicts of interest? We acknowledge that Oren and Garrity followed the ICNP rules correctly in their letter (4), but our and others’ (2, 5) broader argument is that so very few individuals who develop the ICNP rules are also members of the ICSP executive board, and they solely propose changes in nomenclature. We are unsure if the ICSP understands the full extent of opposition to the proposed name changes (https://www.the-scientist.com/news-opinion/newly-renamed-prokaryote-phyla-cause-uproar-69578, https://science.thewire.in/the-sciences/ncbi-taxonomy-prokaryotes-phylum-names-firmicutes-proteobacteria/, https://www.wired.com/story/microbe-names-fight/, https://schaechter.asmblog.org/schaechter/2022/07/a-rose-is-rose-is-a-rose-is-a-roseota.html). Based on this knowledge and our collective observations, we feel that the ICNP rules and their application would benefit from additional scrutiny. Exceptions should be allowed under the current circumstances where, due to overwhelming historical usage, the assigning of phylum names based on the type genus is perplexed. Furthermore, the overall process should change to be much more proactive, aiming to be inclusive of the opinion of practicing microbiologists worldwide, instead of final proclamations made by 21 voting members. We also want to highlight the distribution of these votes (6), as only 10/21 were cast in favor of using genus as the nomenclatural type of a phylum, 7/21 were in favor of using class, and others were of no opinion. It suggests that even ICSP delegates are skeptical about the proposed phylum name-changing rules.

Notably, the ICSP has (un)intentionally not replied to our point 6 (2), where we inquired as to why the name of *Euryarchaeota*, the phylum with the highest representation in the kingdom *Archaea*, was not changed to *Methanobacteriota* (4). If a deviation from the standard practice is allowable in this case, we propose that a similar exception be made for the six phyla under discussion (listed below) to preserve consistency in the literature and not reduce the phylum name to a single representative of a very diverse group.

Oren. et al., 2022 (1) also state that “if Panda et al. believe archaeal phyla should have the suffix *-archaeota*, then a formal proposal to this effect could be made.” We appreciate the ICSP’s guidance and intend to elaborate on such a proposal after we reach out to the global microbiology community. Notably, it would be part of a broader proposal that the ICSP reconsider its changes to six long-established phylum names. In such instances, forcing the ICNP rule of using the type genus names to establish phylum names will lead to unnecessary confusion in the literature. Furthermore, we believe that the proposed ICSP names will create chaos in scientific analyses and the reading, referencing, and comparison of past versus future publications, open-access genomics, and metagenomic data sets.

We certainly support using the *-ota* suffix for all phyla to provide uniformity. However, we propose retaining the long-established root words for the following six phyla to avoid confusion: *Proteobacteria* (new name: *Proteobacteriota*), *Firmicutes* (new name: *Firmicuteota*), *Actinobacteria* (new name: *Actinobacteriota*), *Tenericutes* (new name: *Tenericuteota*), *Crenarchaeota* (same name: *Crenarchaeota*), and *Thaumarchaeota* (same name: *Thaumarchaeota*). In addition, we also recommend that the archaeal phylum name *Euryarchaeota* (same name: *Euryarchaeota*) should be retained. As stated above, we will follow the ICSP advice and make a formal request for an opinion to the ICSP Judicial Commission regarding these phylum names.

We also request that the ICSP implement an email service to which researchers can subscribe to stay informed regarding discussions on rules, nomenclature, and ICSP decisions. Doing so will significantly increase the transparency of these processes, facilitate scientific discourse, and ultimately render nomenclatural decisions even more democratic.
